# Annotations

**Published:** 1901-06-15

**Authors:** 


### Annotations.

### Silk Stockings and Tin Poisoning.

Among the various forms of metallic poisoning to which man is liable, we must not forget that innocent as tin may be as a lining to "tin" vessels, some of its salts are by no means free from poisonous qualities, and when absorbed act seriously upon the nervous system. These salts are often employed in dyeing. Properly used they act as a mordant, but it is whispered that the manufacturer is not always sorry to find that the excess of the salt is not removed, for when left it adds weight to the silk—and silk is valuable—hence many troubles. Coloured stockings have often been accused of causing poisoning. Not only does the pattern on the stocking sometimes cause eruptions on the legs, but in some cases poisonous materials have been absorbed into the system. Arsenic, which in the early days of aniline dyes was often present, used to be the incriminated metal. This, however, is a mode of arsenical poisoning of which very little has been heard for many years. Now it is tin which is arraigned. In producing certain delicate colours in silk chloride of tin is used as a mordant, and in some cases it is said that this salt exists in the dyed fabric in very large proportions. Hence when the fabric takes the form of stockings and the feet perspire, the salt dissolves and is absorbed. A case is reported from Vienna of a woman who suffered from attacks of partial paralysis in the lower extremities, with anæsthesia, a sense of coldness, and ataxic gait. She had noticed that whenever these symptoms were most pronounced her feet were coloured yellow, and it was found that this staining was derived from the light yellow silk stockings which she wore. On analysing these they were found to contain considerable quantities of tin. Careful chemical examination of the excreta showed that they also contained tin, so that whatever may have been the cause of the symptoms there could be no doubt that the patient had tin in her system. The moral seems to be that persons who perspire should not wear pretty silks next their skin unless they can be sure that they are not dyed with colours mordanted with tin. Possibly there may in this case have been some carelessness, and it may not have been the metal combined with the colour but rather the excess, which had not been properly removed, that did the mischief. This, however, will not afford much comfort to the ladies who buy silk stockings, for how are they to tell whether any particular colour is safe or not? It is unfortunate, but it seems to be the fact, that in many instances colours which are "fast" enough in relation to ordinary washing are by no means incapable of solution in perspiration, especially when this natural secretion has been modified by the various fermentive changes which it is apt to undergo.

### The Functions of a University.

In delivering the annual oration in connection with Foundation Week at University College, Professor William Ramsay gave expression to ideas in regard to the functions of a university which, while intended no doubt for the encouragement of those attached to the institution in which he was speaking, incidentally showed how widely the London University had strayed from the ideal. Not teaching alone, certainly not examining alone, or even the giving of rewards, in the form of degrees, for industry in absorbing knowledge—not these but the increase of knowledge are the functions of a university. The tradition of University College had always been, said Professor Ramsay, that it was not merely a place where known facts and theories had been administered in daily doses to young men and women, but that the duty of the professors, assistant professors, teachers, and advanced students, was to increase knowledge. This was the chief function of a university, although not the only one. A university might teach many things, in fact it might be an advanced technical school in various subjects, but it ought to be more, and he believed that its chief work lay in that direction, the watchword of which was "research." This seems to give us at once a clue to what should be the broad distinction between a school or an academy and a university. Professor Ramsay went on to say that there was all the difference in the world between the point of view of the student who read in order to qualify for an examination, or to gain a prize or scholarship, and of one who read because he knew that thus he would acquire what might be used as the basis of new knowledge. It was that spirit in which our universities were so lamentably deficient, while it was that spirit which had contributed to the success of the Teutonic nations, and was beginning to influence the United States. For our deficiencies in this regard our examinational system was largely to blame, a system which had eaten into our education like a canker. He held that a university which did not increase knowledge might be a technical school or a coaching establishment, but it had no claim to the name of university. What, then, we may ask, of the University of London of the past—a mere stamping machine by which the Hall Mark has been attached to those who could pass the assay at a certain standard? Those who are endeavouring to lick the new University of London into shape may well take note of what Professor Ramsay has to say on the functions which a university ought to undertake.

### The Late Mr. Thomas Bond.

The news of the death of Mr. Thomas Bond, consulting surgeon to the Westminster Hospital, who committed suicide last week by throwing himself from his bedroom window, was received with the greatest regret by all who knew him. He had been ill for a considerable time and had suffered much pain, for the relief of which it had been necessary to administer morphia in considerable doses. Evidence was given at the inquest to the effect that, as one result of this drug, he had become very emotional; that his mental power had become affected, and that thus the sad end was to be explained. To those who knew him when in his full vigour the picture thus drawn of his last illness was perhaps even more sad than the news of his death; for when in health he had been the very embodiment of courage, determination, and common sense. He was a man who had borne himself well in many positions, and had made friends under circumstances in which some might have made enemies. Like so many of our leading surgeons he had served as a volunteer in war, having acted as surgeon with the Prussian military forces in 1866. He had been surgeon for many years, and later consulting surgeon, to the Westminster Hospital, where he also was lecturer on forensic medicine, and was well known in connection with the Westminster Training School for Nurses. He was surgeon to the Great Western Railway Company, and professionally was perhaps best known as an adviser in relation to railway injuries. To the public his name was familiar as a police surgeon, and more especially as one whose professional advice was constantly sought in regard to criminal investigations, in which his evidence always commanded the greatest respect.

